# Validity and reliability of a self-report instrument to assess social support and physical environmental correlates of physical activity in adolescents

**DOI:** 10.1186/1471-2458-12-705

**Published:** 2012-08-29

**Authors:** Anne K Reimers, Darko Jekauc, Filip Mess, Nadine Mewes, Alexander Woll

**Affiliations:** 1 Department of Sport Science, University of Konstanz, Universitätsstraße 10, 78457 Konstanz, Germany

**Keywords:** Cross-validation, German, Questionnaire, Motorik-Modul (MoMo), Confirmatory factor analysis

## Abstract

**Background:**

The purpose of this study was to examine the internal consistency, test-retest reliability, construct validity and predictive validity of a new German self-report instrument to assess the influence of social support and the physical environment on physical activity in adolescents.

**Methods:**

Based on theoretical consideration, the short scales on social support and physical environment were developed and cross-validated in two independent study samples of 9 to 17 year-old girls and boys. The longitudinal sample of Study I (n = 196) was recruited from a German comprehensive school, and subjects in this study completed the questionnaire twice with a between-test interval of seven days. Cronbach’s alphas were computed to determine the internal consistency of the factors. Test-retest reliability of the latent factors was assessed using intra-class coefficients. Factorial validity of the scales was assessed using principle components analysis. Construct validity was determined using a cross-validation technique by performing confirmatory factor analysis with the independent nationwide cross-sectional sample of Study II (n = 430). Correlations between factors and three measures of physical activity (objectively measured moderate-to-vigorous physical activity (MVPA), self-reported habitual MVPA and self-reported recent MVPA) were calculated to determine the predictive validity of the instrument.

**Results:**

Construct validity of the social support scale (two factors: parental support and peer support) and the physical environment scale (four factors: convenience, public recreation facilities, safety and private sport providers) was shown. Both scales had moderate test-retest reliability. The factors of the social support scale also had good internal consistency and predictive validity. Internal consistency and predictive validity of the physical environment scale were low to acceptable.

**Conclusions:**

The results of this study indicate moderate to good reliability and construct validity of the social support scale and physical environment scale. Predictive validity was only confirmed for the social support scale but not for the physical environment scale. Hence, it remains unclear if a person’s physical environment has a direct or an indirect effect on physical activity behavior or a moderation function.

## Background

For people of all ages, being physically active is a well-established precondition to health [1-4]. Especially for adolescents, an active lifestyle is necessary for growing up healthy and has been associated with numerous health benefits [5,6]. For example, being regularly physically active increases cardiorespiratory fitness and high-density lipoprotein cholesterol and enhances psychological health [7]. International activity guidelines for adolescents require a minimum level of moderate-to-vigorous physical activity (MVPA) of 60 minutes per day [8,9]. Yet, there is scientific consensus that many adolescents do not engage in a sufficient level of physical activity (PA) [10,11].

Socio-ecological and socio-cognitive models explain health behaviors such as PA and have also been applied to children and adolescents [12]. These models postulate an interaction of individual and environmental factors that influence human health behaviors [13-15]. It has been suggested [16] that environment-changing interventions are important in the field of public health because they may reach a large proportion of the population and achieve sustainable effects. Especially in adolescence, health promotion strategies should consider social and physical environmental aspects [14].

The concept of social support plays an important role in socio-ecological and socio-cognitive theories and is one of the most important social determinants on health behavior. This concept differs conceptually from social norms, modeling, social influence and social networks [17]. In adolescents, the main critical distinction in the social support construct has been made between the different sources or providers of support, respectively. Adolescence is the period of the second separation-individualization process where relationships are getting refined and renegotiated and the social environment changes [18]. While peers may take on a more prominent socially influential role, parents—who were the most important reference persons during childhood—continue to exert considerable influence on adolescents’ behavior. Other reference persons such as grandparents or teachers are less frequent sources of support as children are getting older [19]. As the most important reference persons, parents and peers are the main providers of supportive behavior for adolescents [18]. In addition, the findings of previous studies summarized by various reviews suggest positive effects of parental and peer support on adolescent PA [20-24]. A meta-analysis confirms a significant moderate positive effect of parental behavior on children’s and adolescents’ PA behavior [22].

Furthermore, physical environments provide potential opportunities and barriers to engage in physically active lifestyles [25]. While various models postulate influences of physical environmental aspects on health behaviors, no comprehensive definition of the physical environment has been established. The behavior specific model, the ecological model of active living [15] describes the influences of the environment on active living on multiple levels. The perceived physical environment as one predictive dimension for active living includes the aspects safety, attractiveness, comfort, accessibility and convenience [15].

In the last decade public health research has focused on physical environmental influences in adolescents’ PA [26]. The state of research has been summarized in several reviews [27-29] which have mostly confirmed the impact of the physical environmental dimensions postulated in the ecological model of active living. The most recent review [29] found associations between PA and some variables of the perceived physical environment including walking/biking facilities, traffic speed/volume, unspecified traffic safety and access to recreation facilities. Limstrand [27] identified significant correlations between environmental aspects such as short distance, nearby parks, playgrounds, sidewalks, perceived safety and young people’s use of sports facilities. A meta-analysis on the perceived environment and PA in adults revealed that having shops and services within walking distance explained the greatest amount of variance in PA [25].

While results of previous studies emphasize the importance of social support and physical environmental factors for increasing PA among adolescents, there is a lack of longitudinal and European studies, respectively [21,28]. Appropriate methods for assessing social support and physical environment in adolescents are necessary to bridge this gap. During the last decade, research efforts have increasingly focused on the development of new instruments on this topic [30-37]. However, some of these instruments are very detailed and extensive measurement methods [32,33,36], and most existing questionnaires are in English language and have been developed for use in North-America or Australia [31,32,36]. Because of differences in sports culture (e.g. sports clubs vs. institutionalized sports) and land-use culture (e.g. urban construction), cross-cultural invariance of the instruments can not be expected implicitly [35]. Thus, applying a questionnaire that accounts for the structural and social conditions of the country where a study will take place is critical [35].

Therefore, the development of appropriate, valid and reliable instruments on social support and physical environmental correlates of adolescent’s PA is necessary for longitudinal and European studies. The aim of this study was to assess the psychometric properties of a new German short self-report instrument for measuring social support and physical environmental determinants of PA in adolescents. Specifically, we assessed the construct validity, internal consistency, test-retest reliability and predictive validity.

## Methods

### Design

After questionnaire development, two separate studies were carried out to test the reliability and validity via cross-validation technique. Study I was conducted to estimate the factorial structure, reliability and predictive validity of the instrument. Study II was conducted to confirm the factorial structure of the amended scales that were determined in Study I via confirmatory factor analysis (CFA).

Both studies were approved by the ethics committee of the University of Konstanz and were conducted according to the Declaration of Helsinki.

### Questionnaire development

The instrument on social support and physical environment of PA was specifically developed for use in the “Motorik-Modul” study (MoMo), a longitudinal survey of “Physical Fitness and Physical Activity as Determinants of Health Development of Children and Adolescents” [38]. Items on peer support were derived from the validated scales developed by Ommundsen and colleagues [35], and items on parental support mainly came from a German study on leisure time of adolescents [39]. One item on importance of sports participation was newly developed. Physical environment scales should include a range of items that cover different potential predictors of the physical environment [32]. Therefore, the physical environment scale included three factors (accessibility, convenience and safety) depending on the dimensions of the ecological model of active living [15] as described above. These dimensions have also been identified as the most important physical environment determinants of PA in adults [40] and presumably are also important for adolescents’ PA [27,29]. Safety could be another aspect of the social environment. However, because safety has been part of physical environmental concepts [41] and is intimately linked with multiple characteristics of the physical environment [26], we included the safety dimension in the scale. All items of the physical environment scale were newly developed by an expert group of the MoMo project team. Five seventh-grade students completed the final versions of the scales to test their comprehensibility. The original version of this instrument is available on request from the first author.

### Study I: factorial structure, reliability and predictive validity

#### Participants and data collection

For Study I, a longitudinal sample of 109 boys and 87 girls (*N* = 196) aged between 9 and 17 years (*M* = 12.8; *SD* = 1.6) was recruited from a comprehensive secondary school in Konstanz, Germany. Grade 5 to grade 9 students of all three traditional types of the tripartite German secondary school system were included (Hauptschule: *N* = 28; Realschule: *N* = 63; and Gymnasium: *N* = 105). After receiving informed consent by the participant and their parent, the participants completed the social support and physical environment self-report instrument and the MoMo Physical Activity Questionnaire for adolescents (MoMo-PAQ) twice at a between-test interval of seven days (t_1_ and t_2_). Between t_1_ and t_2_, participants wore an accelerometer and completed the Previous Day Physical Activity Recall (PDPAR) [42] for seven days. The study was conducted on schooldays from April to July 2009.

#### Measures

##### Social support

The social support scale includes two factors that are related to the two main providers of social support: parental support (8 items) and peer support (3 items). The answers are based on a four-point rating scale (e.g. “How important is sport in your family?” 1—“not at all” to 4—“very important”). Higher scores indicated higher perceptions of social support.

##### Physical environment

The factors of the physical environment scale were accessibility of recreation facilities (4 items), convenience (3 items) and safety (4 items). The answers are based on a four-point rating scale (e.g. “In the area I live in, there are sports clubs.” 1—“none” to 4—“many”). Higher scores correspond to a more activity friendly environment.

##### Physical activity

Objective assessments of PA were obtained using the ActiGraph GT1M accelerometer (ActiGraph; Pensacola, FL, USA). The ActiGraph GT1M is a biaxial accelerometer designed to detect vertical and horizontal accelerations ranging in magnitude from 0.05 to 2.00 G’s with a frequency response of 0.25 to 2.50 Hz. The measurement intervals were set to 10-second intervals. Cut points established by Freedson and colleagues [43] were used to calculate adolescents’ MVPA. The Actigraph GT1M has been shown to be a valid and reliable tool for assessing MVPA in children and adolescents [43,44].

The MoMo Physical Activity Questionnaire (MoMo-PAQ) was used to assess self-reported habitual MVPA in adolescents [45]. The MoMo-PAQ consists of 28 items and measures frequency, duration, intensity and setting of PA. Data obtained with the MoMo-PAQ are sufficiently reliable (test-retest reliability: *ICC* = 0.68) and correlates significantly (*r* = 0.29) with accelerometry data [45].

The PDPAR [42] is a self-report measure of PA designed specifically for the cognitive abilities of children and adolescents [46]. In the current study, the PDPAR was used to measure the PA of the preceding week, which was termed ‘recent PA’. A separate table for each day in the week was provided for assessing PA of the preceding day. Each table was divided into several 60-minute time blocks. For each time block, participants could choose one of 38 enumerated activities that were grouped into the following categories: eating, sleeping/bathing, transportation, work/school, leisure time physical activities, and sports. Each activity could be rated according to its intensity (light, moderate, high and very high). The PDPAR has been shown to be a reliable and valid measure of PA of the preceding day in adolescents [47].

#### Statistical analysis

Statistical analyses were performed using PASW statistical software version 18.0 (SPSS Inc., Chicago, IL, USA). In Study I, the amount of missing data was very low for both the social support and physical environment scale (1.8% and 2.0% missing values, respectively). The MCAR-Test by Little [48] was not significant for the social support (χ2 = 121.2; df = 170) and physical environment scale (χ2 = 130.7; df = 121). Because the proportion of missing data was very low and not systematic, we used the method of list wise deletion for handling missing data.

##### Reliability

Internal consistency of the factors was estimated using Cronbach’s alpha and deemed acceptable for values greater than 0.6 [49,50]. The internal consistency measured using Cronbach’s alpha is influenced by the extent of covariation among the items and by the number of items in the scales [49,51]. Hence, there is a trade-off between scale brevity and good reliability. Items that contributed least to the factor’s internal consistency were removed [49]. The seven-day test-retest reliability of the latent factors was assessed using intra-class correlation. ICC estimates above 0.75 were considered as good reliability scores, estimates between 0.50 and 0.75 as moderate reliability scores, and estimates below 0.50 as poor reliability scores, respectively [52]. Reliability calculations were repeated with final factors that were determined using principal components analysis (PCA) as described below.

##### Factorial structure

To determine the factor structure of the instrument, separate PCAs with varimax (orthogonal) rotation were conducted with baseline data of Study I for each of the two scales. Analysis of eigenvalues in the scree plot [53] and the eigenvalue rule [54] were used to determine the number of factors to remain in the given scales. Items were required to have a loading of 0.45 or greater for being assigned to a factor [55]. Otherwise, the scale was modified by removing items with lower factor loadings to achieve better psychometric properties, and the PCA was subsequently re-run [33].

##### Predictive validity

Social support and physical environment are key predictors of adolescents' PA. Thus, we expected a positive correlation between the social support and physical environmental factors and PA behavior. To test the predictive validity of the instrument, different measures of PA were used. Bivariate correlations of the factors (measured at t_1_) with objectively measured MVPA (collected using accelerometry over seven consecutive days), self-reported recent MVPA (seven-day aggregate of PDPAR), and self-reported habitual MVPA (MoMo-PAQ; measured at t_2_) were calculated.

### Study 2: confirmation of construct validity by cross-validation technique

#### Participants and data collection

Construct validity of the questionnaire was examined in Study II. The sample of this study consisted of 430 participants from the German Health Interview and Examination Survey for Children and Adolescents (KiGGS), a representative nation-wide German study [56] conducted from September 2009 to September 2010. The mean age was 13.7 years (*SD* = 1.7 years) and the sample included 221 boys (51.4%) and 209 girls (48.6%). After informed consent was obtained from the participant and their parent, the participants completed the social support and physical environment self-report instrument as described above.

#### Measures

*Social support* and *physical environment* were measured using the same scales as described above for Study I.

#### Statistical analysis

Statistical analyses were performed using AMOS statistical software version 18.0 (SPSS Inc., Chicago, IL, USA). In Study II, the amount of missing data was very low for both the social support scale and the physical environment scale (0.7% and 0.3% missing values, respectively). The MCAR-Test by Little [48] was not significant for the social support (χ2 = 78.3; df = 93) and physical environment scale (χ2 = 57.0; df = 54). Because the proportion of missing data was very low and not systematic, we used the method of list wise deletion for handling missing data.

##### Construct validity

To test the results of the explorative factor analysis of Study I (PCA), a cross-validation was conducted using CFA with the independent data set of Study II. To assess the appropriateness of the models, several indices of fit were used. The χ2statistic assesses absolute fit of the model to the data, is very sensitive to sample size and suggests rejection of the hypothesized model in most cases [57]. The root mean square error of approximation (RMSEA) describes closeness of fit. Values of the RMSEA ≤ 0.06 reflect close and acceptable fit of the model [58]. In addition, if zero is contained in the 90% confidence interval (CI) around the RMSEA point estimate, a good fit is indicated. The Comparative Fit Index (CFI) tests the relative improvement in fit by comparing the proposed model to a baseline model [59]. Values for the CFI around 0.90 were considered acceptable while values around 0.95 indicated a good fit [58,59].

## Results

Descriptive statistics of demographic characteristics and social support scale scores of both studies are summarized in Table 1. Subjects in Study I participated on average in 39.6 min (*SD*=22.2 min) objectively measured MVPA per day and 68.5 min (*SD*=17.2 min) self-reported recent MVPA per day measured by the PDPAR. The self-reported habitual MVPA measured by the MoMo-PAQ was 88.6 min (*SD*=85.5 min) per day.

**Table 1:** Mean ± 1 standard deviation demographics and social support and physical environment factors of Study I and Study II

**Variable**	**Study I (N=196)**		**Study II (N=430)**
	**t**_**1**_	**t**_**2**_	
Age (years)	12.8 ± 1.6	-	13.7 ± 1.7
Sex (boys)	109 (55.6%)	-	221 (51.4%)
BMI (kg/m^2^)	18.8 ± 2.9	-	19.6 ± 3.4
*Physical environment score (possible score range 1.00 – 4.00)*			
Convenience	3.23 ± 0.66	3.13 ± 0.67	3.06 ± 0.75
Public recreation facilities	2.66 ± 0.57	2.73 ± 0.61	2.66 ± 0.67
Safety	3.16 ± 0.53	3.08 ± 0.64	3.30 ± 0.51
Private sport providers	2.25 ± 0.67	2.37 ± 0.70	2.49 ± 0.72
*Social support score (possible score range 1.00 – 4.00)*			
Parental support	2.74 ± 0.61	2.72 ± 0.68	2.92 ± 0.58
Peer support	2.86 ± 0.55	2.76 ± 0.60	2.64 ± 0.58

### Social support scale

Three items were removed from the social support scale (Final versions of the scales are presented in Additional file 1), because they did not contribute to the internal consistency of their factor. According to the Kaiser and scree criterion, the PCA conducted in Study I (Table 2) extracted two factors from the social support scale. These factors describe the constructs of *parental support* and *peer support*. Communalities of the items ranged from 0.47 to 0.73, and the factor loadings ranged from 0.66 to 0.85. Both factors explained 58.2% of variance. The factor structure resulting from the PCA was cross-validated by CFA with the sample of Study II. According to the criteria of Hu [58], the fit of the measurement model in the sample of Study II was good for the two-factor structure of the social support scale (χ2=40.50; df=19; p<0.01; CFI=0.98; RMSEA=0.05 [90% CI=0.03-0.07]).

**Table 2:** Factor structure (varimax rotated solution) and reliability of social support scale (N = 196)

**Factors / items**	***h***^***2***^	**Parental support**	**Peer support**
Do your parents support you in your sports activity (e.g. by buying sporting goods for you)?	*0.47*	**0.69**	0.03
How important is it for your parents that you do sport?	*0.50*	**0.70**	−0.04
How much of an interest do your parents have in your sport?	*0.66*	**0.81**	−0.08
How often is your sport a topic of conversation in your family?	*0.55*	**0.72**	0.17
How often do your parents watch you doing sport?	*0.56*	**0.70**	0.27
How often do you do sport with your friends?	*0.49*	0.24	**0.66**
How often do you ask your friends if they want to play outside or do sport with you (e.g. playing soccer, riding a bicycle, inline skating)?	*0.70*	0.00	**0.84**
How often do your friends ask you if you want to play or do sport with them (e.g. playing soccer, riding a bicycle, inline skating)?	*0.73*	−0.06	**0.85**
**Variance explained** (Overall variance explained =58.2%)		33.7%	24.6%
Rotated eigenvalues		2.69	1.97
Internal Consistency (Cronbach’s alpha)		0.78	0.70
Test-retest reliability (Intra-Class Coefficients)		0.83	0.67

Note: h^2^ = communality; trend of eigenvalues: 2.84; 1.82; 0.75; 0.71; 0.63; 0.53; 0.40; 0.32.

Cronbach’s alphas of both social support factors (α_*parental support*_=0.78; α_*peer support*_=0.70) were acceptable (Table 2). The *parental support* scale had good (*ICC*=0.83) and the *peer support* scale had moderate (*ICC* = 0.67) test-retest reliability, respectively. To assess the predictive validity, Pearson correlations between the *parental support* and *peer support* factors and the three different PA indices were calculated for Study I (Table 3). *Parent support* and *peer support* showed statistically significant positive correlations with all PA indices (range: 0.22–0.31).

**Table 3:** Pearson correlations between PA indices and social support factors of Study I (N = 196)

	**1.**	**2.**	**3.**	**4.**	**5.**
1. Parental support	-	0.17*	0.30**	0.26**	0.22**
2. Peer support		-	0.31**	0.19**	0.28**
3. Self-reported habitual MVPA			-	0.41**	0.29**
4. Self-reported recent MVPA				-	0.37**
5. Objective measured MVPA					-

Note: MVPA = moderate-to-vigorous-physical-activity;

** Correlation is significant at the 0.01 level (2-tailed);

* Correlation is significant at the 0.05 level (2-tailed).

### Physical environment scale

The PCA of the final physical environment scale (Additional file 1) extracted four factors that comprised two items each (Table 4). These factors explained 71.8% of variance. One item was removed from the scale because it lowered the alpha of the factor *safety*. Another two items were removed from the scale because they had a loading of 0.45 or greater on one of the factors [55]. The items belonging to accessibility of recreation facilities loaded on two factors that differentiate between *public recreation facilities* and *private sport providers*. Two other items loaded on the factor *safety* and two on *convenience*. The factor loadings of the physical environmental items ranged from 0.62 to 0.87 and their communalities ranged from 0.49 to 0.79, respectively. The cross-validation with the CFA in Study II showed a good fit for the four-factor model (χ2=32.13; df=14; p<0.01; CFI=0.96; RMSEA=0.06 [90% CI=0.03-0.08]).

**Table 4:** Factor structure (varimax rotated solution) and reliability of physical environment scale (N = 196)

**Factors / Items**	***h***^***2***^	**Convenience**	**Public recreation facilities**	**Safety**	**Private sport providers**
In the area I live in, shops and businesses can be reached on foot.	*0.73*	**0.85**	0.02	0.00	0.13
From where I live, the bus and tram stops can be reached on foot.	*0.77*	**0.87**	0.10	0.02	−0.05
In the area I live in, there are sports facilities that are always accessible (e.g. soccer fields).	*0.70*	−0.06	**0.78**	0.21	0.21
In the area I live in, there are playgrounds.	*0.79*	0.18	**0.87**	−0.02	−0.03
How safe are the public leisure time facilities in the area you live in (in terms of problems with crime)?	*0.74*	−0.05	−0.05	**0.86**	−0.01
For walking and riding a bicycle, the area I live in is … “not very nice at all” to “very nice at all”	*0.49*	0.10	0.32	**0.62**	0.03
In the area I live in, there are sports clubs.	*0.77*	−0.04	0.08	0.32	**0.81**
In the area I live in, there are commercial sport providers (e.g. fitness clubs).	*0.75*	0.13	0.09	−0.32	**0.79**
**Variance explained (**Overall variance explained =71.8%)		19.2%	18.7%	17.1%	16.9%
Rotated eigenvalues		1.53	1.49	1.37	1.35
Internal Consistency (Cronbach’s alpha)		0.64	0.61	0.42	0.48
Test-retest reliability (Intra-Class Coefficients)		0.71	0.74	0.59	0.65

Note: h^2^ = communality; trend of eigenvalues: 1.99; 1.50; 1.24; 1.00; 0.74; 0.55; 0.53; 0.41.

The physical environment factors *convenience* and *public recreation facilities* provided acceptable internal consistency (Table 4). The alphas of *private sport providers* and *safety* were low (α_*private sport providers*_ = 0.48 and α_*safety*_ = 0.42). The one-week test-retest reliability calculated by intra-class correlations was moderate for all factors (Table 4). To assess the predictive validity, Pearson correlations between the factors of the physical environment factors and the three different PA indices were calculated for Study I (Table 5). Only *convenience* was significantly correlated with self-reported habitual MVPA (*r* = 0.24).

**Table 5:** Pearson correlations between PA indices and physical environmental factors of Study I (N=196)

	**1.**	**2.**	**3.**	**4.**	**5.**	**6.**	**7.**
1. Convenience	-	0.15*	0.03	0.11	0.24**	0.07	0.05
2. Public recreation facilities		-	0.23**	0.20**	0.03	−0.02	0.10
3. Safety			-	0.04	0.01	−0.06	0.09
4. Private sport providers				-	0.08	−0.04	−0.12
5. Self-reported habitual MVPA					-	0.41**	0.29**
6. Self-reported recent MVPA						-	0.37**
7. Objective measured MVPA							-

Note: MVPA = moderate-to-vigorous-physical-activity;

** Correlation is significant at the 0.01 level (2-tailed);

* Correlation is significant at the 0.05 level (2-tailed).

## Discussion

The aim of this study was to assess the psychometric properties of a new German short self-report instrument for measuring social support and physical environmental determinants of PA in adolescents. The longitudinal design of Study I allowed for examination of test-retest reliability and predictive validity of the social support and physical environment scales. In addition, the internal consistency and construct validity were assessed.

### Social support

According to theoretical considerations [18], the scale comprised the two factors that characterize the two main providers of social support in adolescents: *parental support* and *peer support*. The two-factorial structure that was identified in Study I using PCA was confirmed using cross-validation technique by performing CFA with the independent nationwide cross-sectional sample of Study II. The CFA showed good model fit for the two factor model which suggests adequate construct validity of this new social support scale. The fit indices of this study were comparable to those reported by Dishman and colleagues [30] for a two-factor model of social support that includes parental and friend support.

The factors *parental support* and *peer support* showed sufficient internal consistency. In comparison with other instruments on social support, the current scale featured comparable internal consistency and test-retest reliability. The alpha estimates were slightly higher than those reported for some other instruments of social support for children [32,34] and adolescents [33,35]. However, Pirasteh and colleagues [37] found a higher internal consistency of a five-item friend support scale in a validation study of a psychosocial determinants questionnaire in Iranian adolescent girls (α = 0.77). Satisfying internal consistency of the brief factor scales suggests that there was a good balance between scale length and reliability. Hence, this scale might be suitable in large-scale health surveys that require short scales.

In this study, the test-retest reliability of the latent factors was moderate to good indicating test-retest-reliability during an interval of one week. In addition, test-retest coefficients were comparable to other instruments that were developed for children and adolescents [30-32,37]. The correlation coefficients were higher for *parental support* than for *peer support*. This finding is consistent with the results of a study by Dishman and colleagues [30] that test-retest reliability for parental support was higher than that for friends support over a two-week interval. This result indicates that either the reliability of the scale for parental support is higher than that of the scale for peer support or that the construct of parental support is more stable over time than the construct of peer support. This discrepancy in reliability between factors may be caused by the fact that the items on *parental support* partly relate to more general perceptions (e.g. “How important is it for your parents that you do sport?”) than the potentially more variable aspects of items on *peer support* (e.g. “How often do you do sport with your friends?”).

Both factors of the social support scale correlated positively with all PA measures (objective MVPA, self-reported recent MVPA and self-reported habitual MVPA) suggesting predictive validity of the instrument. Adolescents who received greater social support from their parents and their peers were more likely to be physically active (measured objectively as well as self-reported) than those who received less social support. The correlation coefficients in this study are comparable to those reported in other studies on the relationship between social support and adolescent PA [22,60]. The highest longitudinal correlations of both parental support and peer support were observed with self-reported habitual MVPA (r_*parental support*_ = 0.30 and r_*peer support*_= 0.31, respectively). The results of this study suggest that social support of parents and peers predicts rather habitual MVPA than recent MVPA (measured objectively as well as self-reported). Because the social support scale contains habitual variables of supportive behavior (e.g. “How often do your parents watch you doing sport?” – “never” to “always”), the higher correlation coefficients may have been caused by the conceptual conformance of the measures of habitual MVPA and habitual social support.

### Physical environment

The CFA showed a good fit for the four-factor model and provides evidence for the construct validity of the physical environment scale. Fit indices were comparable with the indices identified by Ommundsen and colleagues [35] for a model of physical environment containing the three factors opportunity, facility and license. The variability of factor structures across physical environmental questionnaires may be related to the diversity of the construct and its definitions and the lack of behavior-specific theories on physical environmental influences on PA behavior [15,61].

The current scale on the physical environment has a four-factor structure with the latent factors *convenience, public recreation facilities, safety* and *private sport providers*. The items that were developed for measuring accessibility of recreation facilities built upon two separate factors that distinguish between *private sport providers* and *public recreation facilities*. These factors seem to describe different aspects of the physical environment that may offer different opportunities for adolescents for being physically active. It is possible that these items load on two separate factors because the incidence of these facilities is traceable to the population density in the residential area. In Germany, private sports providers are more likely to be located in the center of cities whereas public recreation facilities such as playgrounds or soccer fields may be located in neighborhoods with lower population density and outside of city centers.

The internal consistency of two of the physical environment factors was low which may be related to the brevity of the factors as only two-item measures. However, other validation studies of physical environment instruments on different target populations also showed low internal consistency on the factor level [32,34,35,62], which may be related to the heterogeneity among the physical environment items. Some characteristics of the physical environmental that are relevant for adolescent’s PA may be independent of each other. For example, there is no association between the accessibility of different types of recreation facilities such as soccer fields or playgrounds. Therefore, the physical environment construct may not be appropriately assessed by internal consistency as a measure of reliability [63].

The results of this study showed satisfying one-week test-retest reliability that was comparable to that of other physical environment instruments for children or adolescents [31,32,36]. Nevertheless, it should be noted that most of the dimensions of the physical environment measured here and in the instruments described above are relatively stable local conditions (e.g. access, convenience). Consequently, the limitations of test-retest reliability are likely related to changes in response behavior of participants. Adolescents seem to have varying perceptions of the physical environment or are not able to describe aspects of their physical environment appropriately. Rephrasing items and providing a description of the area of interest (e.g. neighborhood) might improve the participants’ understanding of the questions and consequently increase reliability.

The predictive validity of the physical environment factors could not be confirmed. The correlation coefficients of relationships between the physical environment factors and overall PA indices were in most cases not statistically significant. Only the factor *convenience* positively correlated with self-reported habitual MVPA in adolescents. This result supports findings of Rosenberg and colleagues [36] who reported correlations between land use mix-access (which is a scale of 6 items on access of stores, places and transit stops, parking opportunities, and walking barriers) and routinely walking to shops and routinely walking to parks in adolescents. There is inconclusive evidence on predictive effects of physical environment measures on adolescents’ PA [28] and the concept that the environment has direct effects on behavior is contentious [64]. Hence, the physical environment may be a prerequisite and a moderator of the relationship between psychosocial correlates such as social support and PA behavior in adolescents without a direct effect [65]. In addition, the influence of environmental factors on behavior may develop slowly [64]. The timespan between the measurement points of physical environmental variables and PA behavior in the current study was one week. This period may be too short to detect physical environmental effects. Hence, future research should focus on longitudinal data with longer between-test intervals. Finally, the physical environment should be investigated as a moderator of associations between other PA correlates and adolescents’ PA behavior [65].

### Strength/ limitations

The strength of this study was that the questionnaire was developed based on theoretical considerations and built upon previous instruments. It was cross-validated with two independent study samples of 9 to 17 years old girls and boys. Nevertheless, this study had some limitations. For instance, our considerations were based on two studies with suboptimal sample size. The results of test-retest correlations should be carefully considered because these results are based only on the sample of the Study I with 196 participants. In addition, Study I included a sample of adolescents from only one public school, and hence these students may have had a somewhat similar physical environment. The variability in physical environment variables may have been higher if participants had originated from different areas.

In addition, the instrument has not been validated in a comparison with a 'gold standard' because to date a 'gold standard' for measuring social support or physical environment in adolescents is lacking [34]. Different methods are available for objectively examining the physical environment. However, objectively measured and subjectively measured physical environments do not equally correlate to PA in adolescents [29] and differ by definition, because the objectively measured environment is the actual environment while the subjectively measured environment is the person’s perception of environmental attributes. Hence, future studies could validate the physical environment scale by comparison with objective measurement methods and the social support scale by comparison with proxy-report methods of social support (e.g. parent-report).

## Conclusions

The results of this study provided some evidence on reliability and validity of the social support scale and physical environment scale. Reliability of the two scales is comparable to that of the other existing scales measuring the same constructs. The construct validity of the both scales was confirmed by cross-validation using two independent samples. However, further studies on large samples from diverse neighborhoods and implementing criterion validation techniques are warranted to improve evidence on the measurement properties of the instrument. The predictive validity was only confirmed for the social support scale but not for physical environment scale. In accordance with theoretical considerations and results of other studies [64,65], this issue raises the question whether physical environment has a direct effect on PA behavior or has a moderation function.

## Competing interests

The authors declare that they have no competing interests.

## Authors’ contributions

AKR was responsible for the overall conception and design of this manuscript, statistical analysis and interpretation of data. DJ designed Study I, contributed to the design of Study II, collected the data of Study I, contributed to the statistical analysis and provided edits to the manuscript. FM and NM revised the manuscript. AW designed Studies I and II. All authors read and approved the final manuscript.

## Pre-publication history

The pre-publication history for this paper can be accessed here:

http://www.biomedcentral.com/1471-2458/12/705/prepub

## Supplementary Material

Additional file 1 Final versions of the physical environment and social support scales. Click here for file
