# Supplementary material for: Validity and reliability of a self-report instrument to assess social support and physical environmental correlates of physical activity in adolescents
Source: BMC Public Health. 2012 Aug 29;12:705. doi: 10.1186/1471-2458-12-705 (PMC3489617; doi:10.1186/1471-2458-12-705)
Supplement: Additional file 1 — Final versions of the physical environment and social support scales. [file 1471-2458-12-705-S1.pdf]

Physical environment

1. In the area I live in, there are sports facilities that are always accessible (e.g. soccer fields).

none	few	several	many
❑	❑	❑	❑

2. In the area I live in, there are sports clubs.

none	few	several	many
❑	❑	❑	❑

3. In the area I live in, there are commercial sport providers (e.g. fitness clubs).

none	few	several	many
❑	❑	❑	❑

4. In the area I live in there are playgrounds.

none	few	several	many
❑	❑	❑	❑

5. How safe are the public leisure time facilities in the area you live in (in terms of problems with crime)?

very unsafe	pretty unsafe	pretty safe	very safe
❑	❑	❑	❑

6. For walking and riding a bicycle, the area I live in is

not very nice at all	not that nice	pretty nice	very nice
❑	❑	❑	❑

7. In the area I live in, shops and businesses can be reached on foot

very badly	rather badly	rather well	very well
❑	❑	❑	❑

8. From where I live, the bus and tram stops can be reached on foot

very badly	rather badly	rather well	very well
❑	❑	❑	❑

Social support

9. How often do you do sport with your friends?

never	rarely	often	always
❑	❑	❑	❑

10. How often do you ask your friends if they want to play outside or do sport with you (e.g. playing soccer, riding a bicycle, inline skating)?

never	rarely	often	always
❑	❑	❑	❑

11. How often do your friends ask you if you want to play or do sport with them (e.g. playing soccer, riding a bicycle, inline skating)?

never	rarely	often	always
❑	❑	❑	❑

12. Do your parents support you in your sports activity (e.g. by buying sporting goods for you)?

never	rarely	often	always
❑	❑	❑	❑

13. How often is your sport a topic of conversation in your family?

never	rarely	often	always
❑	❑	❑	❑

14. How important is it for your parents that you do sport?

not important at all	a little important	pretty important	very important
❑	❑	❑	❑

15. How much of an interest do your parents have in your sport?

none at all	a little bit	pretty strong	very strong
❑	❑	❑	❑

16. How often do your parents watch you doing sport?

never	rarely	often	always
❑	❑	❑	❑
